# Complete chloroplast genome sequence of *Acer ginnala*, an important ornamental tree

**DOI:** 10.1080/23802359.2019.1710606

**Published:** 2020-01-14

**Authors:** Hongxin Yang, Xi Zha, Shilin Cao, Ying Wang, Fei Gao, Yijun Zhou

**Affiliations:** College of Life and Environmental Sciences, Minzu University of China, Beijing, People’s Republic of China

**Keywords:** *Acer ginnala*, chloroplast genome, phylogenetic analysis

## Abstract

*Acer ginnala* is a woody Acer plant with high ornamental value. In the present study, the chloroplast genome of *A. ginnala* was determined, annotated, and analyzed phylogenetically. The total chloroplast genome was 156,184 bp in length, consisting of a large single-copy region (86,525 bp), a small single-copy region (18,947 bp), and two inverted repeat regions (25,356 bp). The complete chloroplast genome contains 133 genes, including 90 protein-coding genes, 36 transfer RNA (tRNA) genes, and 8 ribosomal RNA (rRNA) genes. The phylogenetic analysis based on the sequences of 49 common proteins from 25 species demonstrated a close relationship between *A. ginnala* and three others Acer plants species including *A. truncatum, A. miaotaiense*, and *A.catalpifolium.* This study will help to understand the phylogenetic position of *A. ginnala* in genus Acer.

The genus *Acer*, commonly known as maple, consists of many common deciduous trees and shrubs distributed in the Northern Hemisphere (Ogata 1967). There are more than 200 *Acer* species in the world, mainly distributed in the temperate regions of Asia, Europe, and North America (Harris et al. 2017). Many plants in genus *Acer* have high ornamental value and *Acer* plants are widely used in garden applications, serving as hedges, small sidewalk screens, or shaped trees. In addition, many *Acer* plants have been found to contain a large number of phytochemicals that have antioxidant, antitumor, or anti-inflammatory activities (Choi et al. 2005), thus might be used to produce herbal medicine for treating disease in the future.

The chloroplast genome is highly conserved among plant species and can provide important molecular data for phylogenetic research, biogeographical research, and genetic diversity research (Abla et al. 2019). At present, chloroplast sequencing and annotation of several *Acer* plants have been conducted, including *A. cinnamomifolium* (Chen, Zhang et al. 2019), *A. truncatum* (Chen, Liu et al. 2019), and *A. buergerianum* (Xu et al. 2017). In the present study, the complete chloroplast genome of *A. ginnala* was assembled, annotated, and analyzed phylogenetically.

The leaf sample of *A. ginnala* was collected from Beijing Botanical Garden, Beijing, China (106°79′E, 39°83′N). The leaf sample (20190816-07) was stored in the College of Life and Environmental Sciences, Minzu University of China, Beijing. Genomic DNA was extracted from fresh leaves using the modified CTAB method (Doyle 1987). Small fragment library (350 bp) was constructed and sequenced using Illumina Hiseq2500 platform. Finally, approximately 10 GB clean reads (paired-end 150 bp) were generated. The chloroplast genome was assembled with NOVOPlasty v 3.7.2. (Dierckxsens et al. 2017) and annotated with GeSeq.

The complete chloroplast genome of *A. ginnala* was 156,184 bp in length (GenBank accession: MN_790641), containing a large single-copy region (86,525 bp), a small single-copy region (18,947 bp), and a pair of inverted repeat regions (25,356 bp). A total of 133 unique genes were annotated from the chloroplast genome of *A. ginnala* including 90 protein-coding genes, 8 ribosomal RNA genes, and 36 tRNA genes. The GC content of the chloroplast genome of *A. ginnala* is 37.97%.

To investigate the phylogenetic position of *A. ginnala*, 24 complete chloroplast genomes were download from NCBI GenBank and then animo acid sequences of 49 common proteins from the 24 plant species and *A. ginnala* were extracted and used for phylogenetic analysis. The sequences alignment was performed using MAFFT v7.450 (Katoh and Standley 2013) and a phylogenetic tree was constructed by RAxML (Stamatakis 2014) using maximum likelihood algorithm. The results showed that most of the nodes in the phylogenetic tree have been strongly supported and all the 12 *Acer* plants were clustered into a single clade. *Acer ginnala* has a close relationship between *A. ginnala* and three others plant species including *A. truncatum, A. miaotaiense*, and *A.catalpifolium.* Our study will help to understand the phylogenetic position of *A. ginnala* in genus *Acer* (Figure 1).

**Figure 1.**
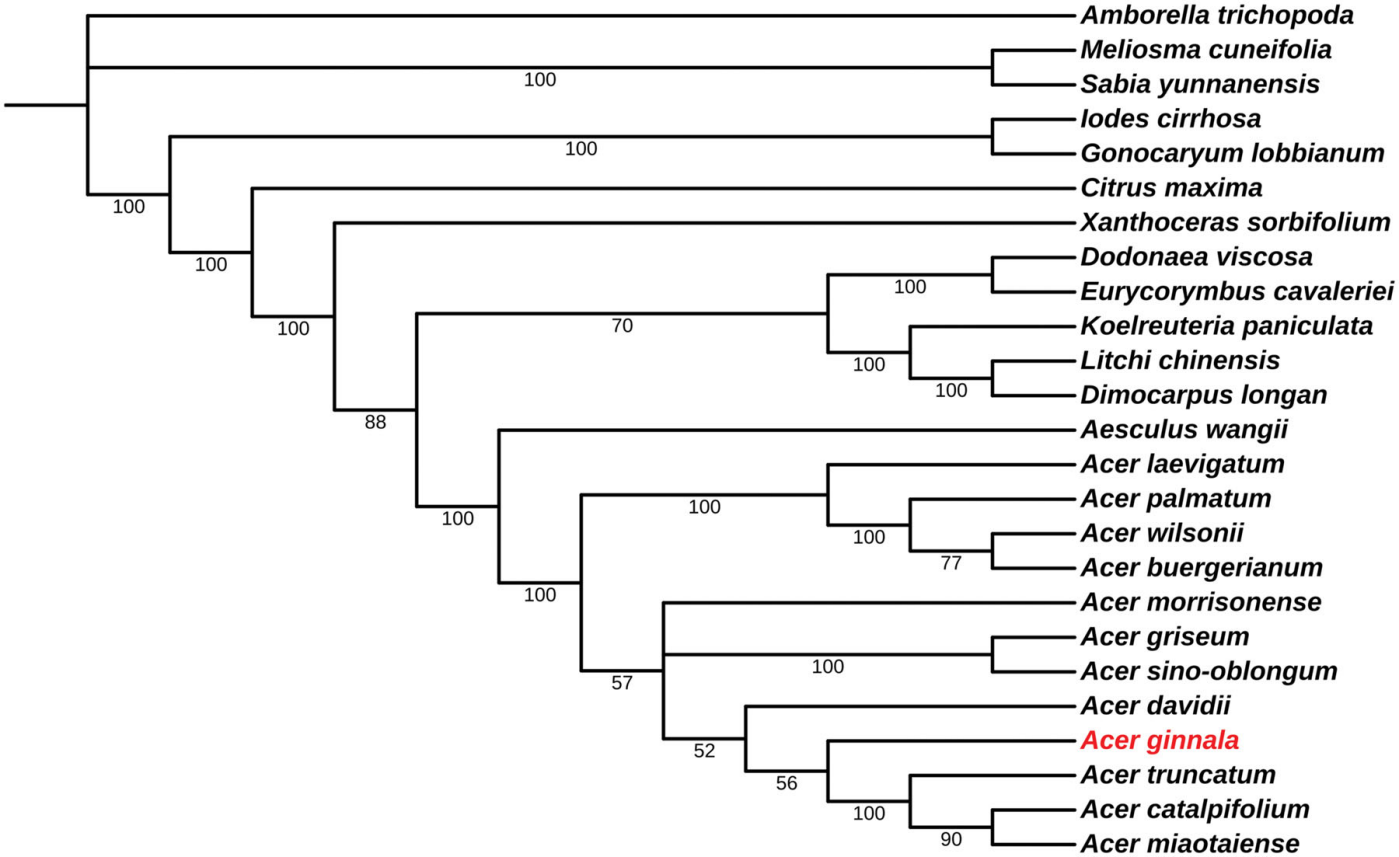
A phylogenetic tree constructed based on 25 complete chloroplast genome sequences of Sapindales. Bootstrap support is indicated for each branch. GenBank accession numbers: *Amborella trichopoda* (NC_005086.1), *Meliosma cuneifolia* (NC_029430.1), *Sabia yunnanensis* (NC_029431.1), *Iodes cirrhosa* (NC_036304.1), *Gonocaryum lobbianum* (NC_041492.1), *Citrus maxima* (NC_034290.1), *Xanthoceras sorbifolium* (NC_037448.1), *Dodonaea viscosa* (NC_036099.1), *Eurycorymbus cavaleriei* (NC_037443.1), *Koelreuteria paniculata* (NC_037176.1), *Litchi chinensis* (NC_035238.1), *Dimocarpus longan* (NV_037447.1), *Aesculus wangii* (NC_035955.1), *Acer laevigatum* (NC_042443.1), *Acer palmatum* (NC_034932.1), *Acer wilsonii* (NC_040988.1), *Acer buergerianum* (NC_034744.1), *Acer morrisonense* (NC_029371.1), *Acer griseum* (NC_034346.1), *Acer sino-oblongum* (NC_040106.1), *Acer davidii* (NC_030331.1), *Acer ginnala* (MN_790641), *Acer truncatum* (NC_037211.1), *Acer catalpifolium* (NC_041080.1), and *Acer miaotaiense* (NC_030343.1).
